# Tumour-induced Osteomalacia Secondary to a Sarcoma

**DOI:** 10.17925/EE.2016.12.02.104

**Published:** 2016-08-28

**Authors:** Karla Victoria Rodriguez-Velver, María Azucena Zapata-Rivera, Juan Montes-Villarreal, Fernando Javier Lavalle-Gonzâlez, José Gerardo González-González, Jesús Zacarías Villarreal-Pérez, Rene Rodríguez-Gutierrez

**Affiliations:** 1. Endocrinology Division, University Hospital “Dr. Jose E. Gonzalez”, Medical School, Autonomous University of Nuevo Leon, Monterrey, Mexico;; 2. Knowledge and Evaluation Research Unit in Endocrinology, Mayo Clinic, Rochester, Minnesota, US;; 3. Division of Endocrinology, Diabetes, Metabolism and Nutrition, Mayo Clinic, Rochester, Minnesota, US

**Keywords:** Tumour-induced osteomalacia, hypophosphatemia, fibroblast growth factor 23

## Abstract

Tumour-induced osteomalacia (TIO), is a rare paraneoplasatic syndrome found in >95% of benign tumours that secrete fibroblast growth factor 23 - a phosphaturic circulating hormone. A rare case of a TIO secondary to a sarcoma, in a 21-year old man with history of bone fractures and distinctive physical and biochemical characteristics is presented and discussed.

Oncogenic osteomalcia, also known as tumour-induced osteomalacia (TIO), is a rare paraneoplasatic syndrome with around 350 reported cases.^1^ TIO initial symptoms are nonspecific and include fatigue, bone pain, muscle weakness, weight, and height loss, and later bone deformity and fractures. It is characterised by hypophosphatemia, hyperphosphaturia, elevated alkaline phosphatase, and low serum 1-25(OH) vitamin D. Pathophysiologically, TIO is caused by tumours that secrete fibroblast growth factor 23 (FGF-23), a phosphaturic circulating hormone.

TIO tumours are usually benign (in >95% of cases), however, they are a diagnostic challenge owing to their small size (usually less than 1 cm), which makes them difficult to localise. The differential diagnoses are genetic causes such as X-linked hypophosphatemic rickets, autosomal dominant and recessive hypophosphatemia, Dent’s disease, idiopathic hypercalciuria, and hereditary hypophosphatemic rickets with hypercaliuria.^2^ Ten cases of TIO in the literature have been reported to be secondary to sarcomas, however, all of them were reported before it was known that the secreted molecule by these tumours was FGF-23, hence the diagnosis remained ‘uncertain’. Herein, we report the case of a 21-year-old male with a TIO associated with spindle cell sarcoma.

## Case report

**Figure 1:**
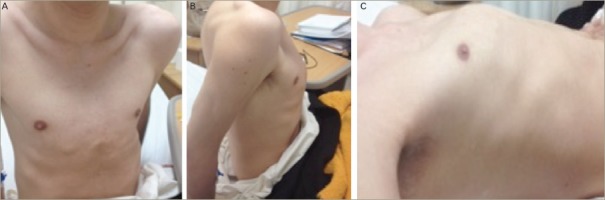
Physical examination A: Sternum protrusion frontal view; B: kyphosis, lateral view; C: Sternum protrusion in supine position.

A 21-year-old man presented for evaluation of a pathological femur fracture. The patient had been otherwise healthy until age 18, when he noted onset of lower back pain after soccer practice, which was exacerbated by exercise and improved when being resting. This pain was progressive and he started noticing a loss of height. Over the next several months he experienced sudden, intense lower back pain and was diagnosed with a femur neck fracture. He had surgery with internal fixation, and stayed mainly in bed for the next 6 months. During this time, he recognised a growing tumour (around 2 cm) in the inner thigh of his right leg. This tumour progressively grew until it was around 4 x 10 cm. During this time he also lost height (going from 1.75 m to 1.65 m) and weight (losing 15–20 kg in total), and noticed kyphosis in the thoracic spine and sternum protrusion. While he was walking, he fell and suffered a pathologic right femoral fracture. On physical examination, his vital signs at presentation were normal and he was noted to have kyphosis, sternum protrusion, and no Harrison’s sulcus or pain at rib palpation (see *Figure 1*). Interestingly, a 4 x 10 cm tumour was noticed in the inner thigh of his right leg (rigid consistency, with rough edges, partially mobile and not painful). The neurologic evaluation and strength were unremarkable.

**Figure 2:**
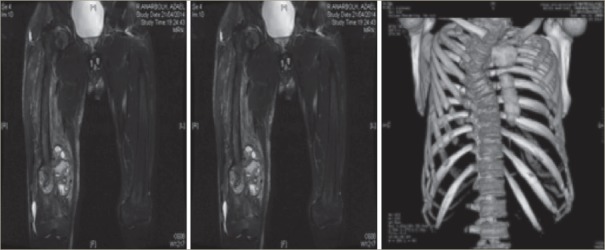
Magnetic resonance scanning T1 Image showing 9 cm tumor in medial right femur. Chest image reconstruction.

A biochemical evaluation (*Table 1*) was noteworthy for severe hypophosphatemia associated with hyperphosphaturia and reduced tubular reabsorption of phosphorus (0.5 g in 24-hour collection), low serum 1, 25(OH)^2^ D, increased serum alkaline phosphatase (ALP). The serum 25(OH) D levels were low and the FGF-23 was 389 RU/m. The remainder of the chemistry profile was within normal limits, including the serum calcium and intact parathyroid hormone (PTH). A diagnosis of hypophosphatemic osteomalacia due to a tumour was made. The magnetic resonance scanning demonstrated a 9 cm mass in the medial right femur, which involved bone and soft tissue (see *Figure 2*).

Resection of the lesion revealed a spindle cell sarcoma with areas of necrosis and nuclear atypia that showed tendency to collagenation, and although it did not form malignant osteoid, cells were positive in immunohistochemistry for osteonectin, which suggests osteoblastic and osteogenic origin with high-grade fibroblastic osteosarcoma being the best fit option, and whose association with hyperphosphaturia is very rare. The neoplasm was in touch with resection borders.

After surgery the patient persisted with low levels of phosphorus, and adjuvant chemotherapy was started with six cycles of adriamycin and cisplatin, with a consequent normalisation of phosphorus levels (2.7 mg/dl).

## Discussion

The first patient with TIO was reported in 1947 by McCance^3^ who attributed cure to high-dose vitamin D treatment. In 1964, in a similar case, Dent and Friedman^4^ described spontaneous recovery after surgical enucleation of the tumour and recognised the link between the neoplasm and metabolic bone disease. In 1959, Prader et al.^5^ were the first to recognise tumour (a giant cell reparative granuloma of the rib) as a cause of osteomalacia. Most of these cases were benign TIO and belong to the same histopathologic entity of phosphaturic mesenchymal tumour (PMC). In fact, Folpe,^6^ in a comprehensive review of the literature found that most of the cases reported as other pathologies were secondary to these. There have been, however, reported cases of malignant tumours such as adenocarcinoma of the colon,^7^ prostate cancer, metastatic tumours, as well as oseteosarcoma.^8–12^

**Table 1:** Baseline and after surgery paraclinical results

Serum values	Baseline	After surgery	Normal range
Hemoglobin (g/dL)	12.1	11.5	12.2–15–5
Sodium (mmol/L)	140.0	138.0	135.0–145.0
Potassium mmol/L)	3.5	3.4	3.5–4.5
Calcium (mg/dl)	9.2	8.9	8.4–10.2
Phosphorus (mg/dl)	0.8	1.2	2.5–4.6
Magnesium	1.8	1.8	1.8–2.5
Alkaline phosphatase (IU/I)	284.0	317.0	38.0–126.0
Creatinine (mg/dl)	0.6	0.8	0.6–1.4
intact PTH (pg/ml)	55.6	60.0	15.0–65.0
1,25(OH)D (pg/ml)	22.8		10.0–75.0
25(OH)D (ng/ml)	11.5		30.0–100.0
pH	7.36	7.38	7.35–7.45
Urine values			
Calcium (mg/24 hrs)	117.76	98.0	100–300 mg/24 hrs
Phosphorus (g/24 hrs)	0.5	0.46 g/24 hrs	0.4 – 1.3 g/24 hrs
Fibroblast growth factor 23	389	72.0	44–205 RU/m

It is important to differentiate our case of malignant presentation PMC^13–15^ since histologically fusiform cells present invasion, which differentiate them from mesenchymal tumours. In this case, unlike the previous reported osteosarcoma, we had levels of FGF-23 that decreased after surgery, however, they will not be normalised until after treatment with chemotherapy. Because of the high recurrence risk in these tumours, it is important to keep monitoring this patient (that is, initially with serum and urine phosphorus).

FGF-23 inhibits the NaPO4 renal co-transporter and also suppresses 1α-hydroxylase activity, resulting in decreased renal reabsorption and increased urine excretion of the phosphate. When a tumour is surgically unresectable or not localised after an extensive workup, oral combined with phosphate treatment, calcium, and calcitriol is recommended, along with phosphate monitoring, a treatment similar to that used in the genetics forms (autosomal dominant hypophosphatemic rickets [ADHR], X-linked). In our case, the tumour was relatively easy to find. Most tumours are found in the extremities, but conventional X-ray, magnetic resonance imaging (MRI), and computed tomography (CT) scans are usually not sufficient for tumour identification and localisation. Because these tumours typically express somatostatin receptors, functional imaging with radiolabeled somatostatin analogs (for example, 111-Indium octreotide scintigraphy or [68Ga] DOTATATE-PET [preferably in combination with CT]) is probably the most valuable diagnostic tool. The treatment of TIO is resection of the tumour and the symptoms and phosphorus levels return to normal. If the tumour is not found, usually we must continue with medical treatment with phosphorus supplementation and vitamin D. Overall, the tumour prognosis is good, however, in our patient, due to the diagnosis of sarcoma, close follow-up will be mandatory.

## Conclusion

TIO, which is currently not frequently suspected, generally occurs when the disease is advanced and the patient has experienced multiple fractures and other complications. Usually they are benign tumours however, as in our case, a small proportion of them can be malignant.
